# Urinary Metabolites Enable Differential Diagnosis and Therapeutic Monitoring of Pediatric Inflammatory Bowel Disease

**DOI:** 10.3390/metabo11040245

**Published:** 2021-04-15

**Authors:** Mai Yamamoto, Meera Shanmuganathan, Lara Hart, Nikhil Pai, Philip Britz-McKibbin

**Affiliations:** 1 Department of Chemistry and Chemical Biology, McMaster University, Hamilton, ON L8S 4M1, Canada; yamamoto.mai88@gmail.com (M.Y.); shanmm2@mcmaster.ca (M.S.); 2 Department of Pediatrics, Division of Pediatric Gastroenterology and Nutrition, McMaster University, Hamilton, ON L8S 4K1, Canada; lara.hart@medportal.ca (L.H.); pain@mcmaster.ca (N.P.); 3 Farncombe Family Digestive Health Research Institute, McMaster University, Hamilton, ON L8S 4K1, Canada

**Keywords:** metabolomics, pediatric inflammatory bowel disease, Crohn’s disease, ulcerative colitis, urine, biomarker discovery, diagnosis, exclusive enteral nutrition, treatment monitoring, capillary electrophoresis-mass spectrometry

## Abstract

Rates of pediatric Crohn’s disease (CD) and ulcerative colitis (UC) are increasing globally. Differentiation of these inflammatory bowel disease (IBD) subtypes however can be challenging when relying on invasive endoscopic approaches. We sought to identify urinary metabolic signatures of pediatric IBD at diagnosis, and during induction treatment. Nontargeted metabolite profiling of urine samples from CD (*n* = 18) and UC (*n* = 8) in a pediatric retrospective cohort study was performed using multisegment injection-capillary electrophoresis-mass spectrometry. Over 122 urinary metabolites were reliably measured from pediatric IBD patients, and unknown metabolites were identified by tandem mass spectrometry. Dynamic changes in sum-normalized urinary metabolites were also monitored following exclusive enteral nutrition (EEN) or corticosteroid therapy (CS) in repeat urine samples collected over 8 weeks. Higher urinary excretion of indoxyl sulfate, hydroxyindoxyl sulfate, phenylacetylglutamine, and sialic acid were measured in CD as compared to UC patients, but lower threonine, serine, kynurenine, and hypoxanthine (*p* < 0.05). Excellent discrimination of CD from UC was achieved based on the urinary serine:indoxylsulfate ratio (*AUC* = 0.972; *p* = 3.21 × 10^−5^). Urinary octanoyl glucuronide, pantothenic acid, and pyridoxic acid were also identified as specific dietary biomarkers of EEN in pediatric IBD patients who achieved clinical remission. This work may complement or replace existing strategies in the diagnosis and early management of children with IBD.

## 1. Introduction

Crohn’s disease (CD) and ulcerative colitis (UC) carry a significant global disease burden [1]. Approximately 20–30% of patients with inflammatory bowel disease (IBD) experience their first symptoms before the age of 18 years. Childhood-onset IBD tends to have a more aggressive phenotype than adult-onset disease, and their unpredictable disease course also impacts normal growth and development [2], educational attainment, and mental health [3]. Early onset, higher severity disease also coincides with a greater utilization of healthcare resources due to accrued complications of surgical involvement, poor nutrition, and medication side effects [4]. Differentiating between CD and UC is critical to optimizing treatment approach [5,6]. For example, exclusive enteral nutrition (EEN) is first-line treatment for induction of remission in children with CD as it has not been proven effective in pediatric UC [7]. Endoscopic assessment under general anesthesia with tissue biopsy and magnetic resonance enterography are invasive, resource-intensive, and costly, but remain standard-of-care to maximize diagnostic accuracy [8]. Nevertheless, neither modality leads to conclusive results in all cases, with pediatric patients prone to higher rates of indeterminate colitis, or IBD-unclassified [9]. These diagnostic dilemmas reflect the poorly understood etiology of IBD in children, particularly early onset IBD, which is mediated by a complex interplay of genetic, immunological and environmental factors.

Nontargeted metabolic phenotyping provides new mechanistic insights into chronic diseases of complex etiology, including the assessment of bioactive compounds from habitual diet, environmental exposures, and commensal microbial activity [10]. To date, metabolomic studies have largely focused on comparing metabolic profiles between IBD patients and healthy controls when analyzing blood, urine, stool and/or colonic mucosa specimens [11,12]. Recently, a time series multiomic gut microbiome study revealed that the serum metabolome is generally less diverse among IBD patients as compared to healthy controls, with several metabolites associated with IBD-related dysbiosis [13]. Previous studies have also reported changes in microbial derived metabolites in IBD patients [14,15], as well as alterations in amino acids, lipids and organic acids reflecting impairments in energy homeostasis due to poor nutrient absorption [16,17,18]. In contrast to distinctive metabolic phenotype differences between healthy controls and patients with IBD or irritable bowel syndrome [19], differentiation between CD and UC has proven far more elusive with often contradictory results [20,21]. Indeed, confounding is more likely among adult IBD patients with a long history of surgical treatment(s) and pharmacological management. In contrast, metabolomic studies involving recently diagnosed and treatment naïve pediatric IBD patients has been largely unexplored to date [12]. Newly diagnosed pediatric IBD patients may offer greater insights into disease pathogenesis, while greater diagnostic certainty can ensure appropriate, targeted treatments are initiated earlier.

In this retrospective cohort study, the urine metabolome is characterized from a cohort of pediatric IBD patients who subsequently receive EEN or corticosteroid (CS) induction therapy. We recently demonstrated that changes in gut microbiome diversity is a key factor associated with mucosal healing and clinical remission, independent of treatment modality and IBD disease subtype [22]. In the present work, a panel of urinary metabolites are identified to differentiate pediatric CD from UC that is not feasible by conventional inflammatory biomarkers in stool and serum. Specific urinary metabolites can also serve to monitor children for adherence to EEN as compared to CS induction therapy when using a validated metabolomics data workflow for biomarker discovery [23]. This report introduces a noninvasive approach to the diagnosis and management of recently diagnosed pediatric IBD patients.

## 2. Results

### 2.1. Study Population and Pediatric IBD Characteristics

Most pediatric IBD patients (*n* = 26) in this sex and age-balanced cohort (mean age of 13 years) were newly diagnosed cases (~73%, <1 month) with only 27% receiving maintenance medications at time of urine collection (Table 1). All patients had elevated serum C-reactive protein (CRP) and fecal calprotectin (FCP) levels at study entry indicating systemic and colonic inflammation, respectively. IBD diagnosis and CD/UC classification was determined by combined endoscopic findings, histopathology and magnetic resonance enterography findings. In this study, 15 of 18 patients with CD received EEN induction therapy, whereas 7 of 8 patients with UC received CS induction therapy. Overall, 57% of IBD patients achieved full clinical remission, and 32% showed a clinical response with significantly lower inflammation after 8 weeks of EEN or CS therapy as compared to baseline.

### 2.2. Urine Metabolome of Pediatric IBD Patients and Metabolite Authentication

**Figure 1:**
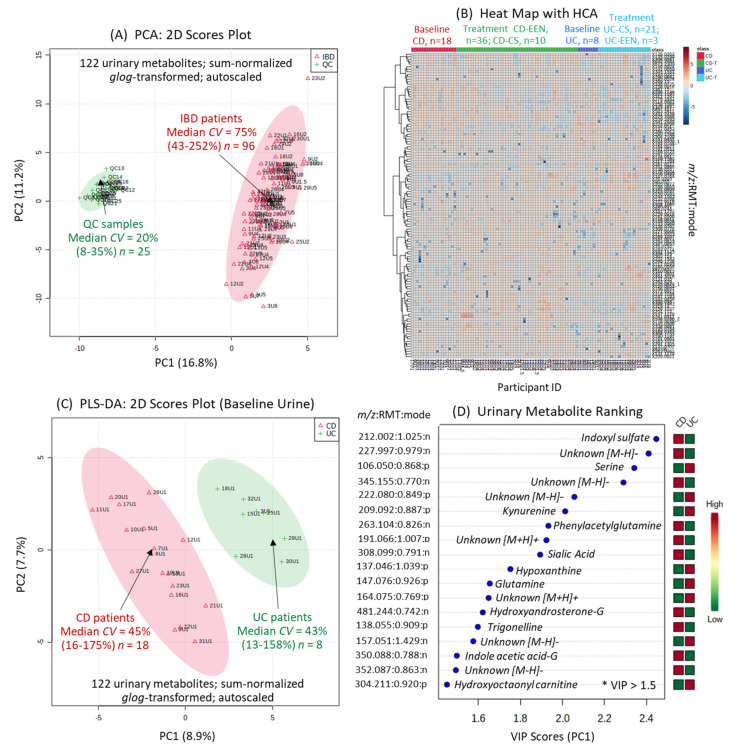
The urine metabolome of pediatric IBD patients by MSI-CE-MS prior to and following induction therapy via exclusive enteral nutrition (EEN) or corticosteroid (CS) intervention. (**A**) A 2D scores plot using principal component analysis (PCA) highlighting the technical precision (pooled urine as QC samples) as compared to biological variance of 122 metabolites measured consistently (CV < 35%) in most urine samples (>75%). (**B**) A 2D heat map with hierarchical cluster analysis (HCA) provides an overview of the data structure and study design, including baseline urine metabolic phenotypes for Crohn’s disease (CD) (*n* = 18) and ulcerative colitis (UC) (*n* = 8) patients who were mainly treated by EEN and CS induction therapy over 8 weeks, respectively. (**C**) Supervised multivariate data analysis of the urine metabolome from pediatric IBD patients at baseline when using partial least squares-discriminate analysis (PLS-DA) enables differentiation of CD from UC affected children based on (**D**) 18 top-ranked urinary metabolites having variable importance in projection (VIP) scores > 1.5. Leave-one-out cross-validation was performed on the PLS-DA model using five components.

A targeted and nontargeted approach for urine metabolic phenotyping of pediatric IBD patients was performed using multisegment injection capillary electrophoresis-mass spectrometry (MSI-CE-MS) as a high throughput platform that is optimal for the analysis of polar/ionic metabolites from volume-restricted biospecimens [24]. A dilution trend filter in MSI-CE-MS was used for molecular feature selection and metabolite authentication in a pooled urine sample from pediatric IBD patients (that also served as a quality control, QC) while rejecting spurious ions, degenerate signals, and background compounds [25]. Mean responses for all urinary metabolites were normalized to an internal standard, and annotated based on their accurate mass, relative migration time and detection mode (*m*/*z*:RMT:mode). A total of 132 urinary metabolites (66 cations, 66 anions) were fully authenticated using this approach (Supplementary Table S1), and the majority (>65%) were identified with high confidence after spiking pooled urine with authentic standards (level 1). Otherwise, urinary metabolites were putatively identified based on collision-induced dissociation experiments with MS/MS spectral comparisons to public databases (level 2), or the annotation of distinctive fragment ions and neutral losses when reference spectra were lacking (level 3). Overall, a final list of 122 urinary metabolites were evaluated after excluding less frequently detected metabolites (<75% of urine samples) and/or compounds having poor technical precision as measured in QC samples (CV > 35%). For instance, several rejected ions included specific drugs and their urinary metabolites (e.g., acetaminophen sulfate), including propofol glucuronide (level 3) since propofol is administered intravenously as a general anesthetic for pediatric endoscopy at baseline (Supplementary Figure S1). Similarly, urinary mesalamine or 5-aminosalicylic acid was detected in only a subset of IBD patients prescribed this maintenance medication prior to recruitment (Supplementary Table S1). In contrast, phenyl sulfate is an endogenous urinary metabolite that is measured consistently with good technical precision in most urine samples (Supplementary Figure S2). Figure 1A depicts a 2D scores plot when using principal component analysis (PCA), which compares the biological variance of 122 sum-normalized urine metabolites from pediatric IBD patients (median CV = 75%, *n* = 96) relative to the technical precision from QC samples (median CV = 20%, *n* = 25). Figure 1B also shows a 2D heat map of the urine metabolome in this longitudinal study involving two IBD subtypes at baseline (CD; UC), with dynamic changes following EEN or CS therapy (CD-EEN; UC-CS) over 8 weeks. Few CD patients crossed-over to CS therapy (CD-CS) at the discretion of the treating gastroenterologist if they did not show adequate response to EEN therapy.

### 2.3. Urinary Biomarker Candidates Which Differentiate Pediatric CD from UC 

**Figure 2:**
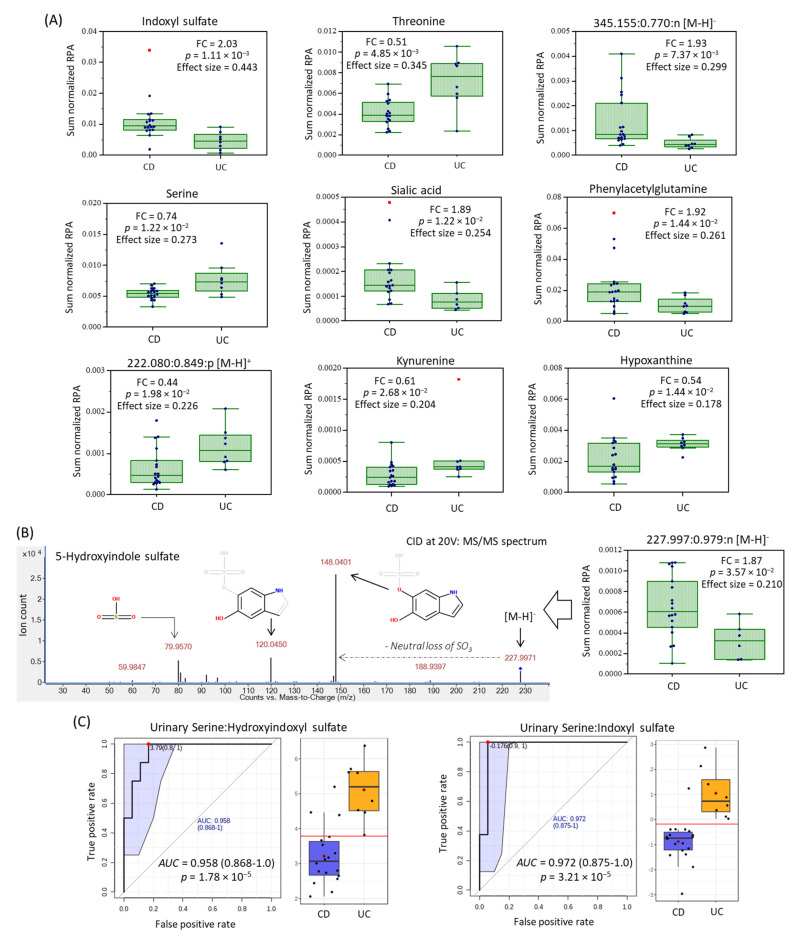
Top-ranked urinary metabolites that differentiate CD from UC pediatric patients (*p* < 0.05) following sum (or osmolality) normalization when using a Mann–Whitney *U*-test. (**A**) Box-whisker plots for 10 urinary metabolites, including unknown metabolites annotated based on their characteristic accurate mass, relative migration time and ion detection mode (*m*/*z*: RMT:mode) were subsequently identified by collision induced dissociation with MS/MS, as shown for (**B**) 5-hydroxy indoxyl sulfate. (**C**) Receiver operating characteristic (ROC) curves are depicted for two top-ranked ratiometric urinary biomarkers following *glog* transformation with good discrimination potential for the two major subtypes of pediatric IBD in affected children (*AUC* > 0.960–0.970, *p* < 3.2 × 10^−5^).

A baseline comparison of the urine metabolome between CD and UC was next performed using complementary multivariate and univariate data analysis. Figure 1C shows that partial least squares-discriminant analysis (PLS-DA) can differentiate CD (*n* = 18) from UC (*n* = 8) patients upon recruitment as shown in the 2D scores plot that demonstrates good model performance following leave-out-one cross-validation (*R*^2^ = 0.996, *Q*^2^ = 0.335). Figure 1D highlights that 18 urinary metabolites mainly responsible for discriminating CD from UC in the PLS-DA model, which were ranked based on their variable importance in projection (VIP > 1.5), including several unknown ions. However, only 10 urinary metabolites were found to consistently differentiate CD from UC children based on either sum (Table 2) or osmolality normalized (Supplementary Table S2) data when using a Mann–Whitney *U*-test (*p* < 0.05). Figure 2A depicts box-whisker plots for top-ranked urinary metabolites from recently diagnosed children with IBD, where CD is characterized by higher excretion of tryptophan or phenylalanine catabolites as compared to UC, including indoxylsulfate, phenylacetylglutamine, and an unknown indole metabolite subsequently identified (level 3) as 5-hydroxyindoxyl-3-*O*-sulfate (Figure 2B). The MS/MS spectrum of this previously unreported oxidized indole metabolite shows a neutral loss for sulfur trioxide (*m*/*z* 79.957) with formation of dihydroxyindole as the base peak/product ion (*m*/*z* 148.0401). As expected, hydroxyindoxyl sulfate was also strongly correlated with urinary indoxyl sulfate (*r* = 0.594, *n* = 97) in pediatric IBD patients. Additionally, urinary sialic acid (*N*-acetylneuraminic acid) and a singly charged unknown anion (345.155:0.770:n; C_16_H_26_O_8_) suggestive of a modified dicarboxylic acid (Supplementary Figure S3), were higher in CD as compared to UC children. On the other hand, children with UC excreted higher levels of serine, threonine, kynurenine, hypoxanthine, and an unknown sulfur-containing singly charged cation (222.078:0.849:p, C_8_H_15_NO_4_S) tentatively identified (level 3) as carboxybutylhomocysteine (Supplementary Figure S4); the latter metabolite was moderately correlated (*r* = 0.566) with urinary hypoxanthine. However, authentic standards were unavailable for direct spiking preventing unambiguous structural elucidation of these unknown ions, including their exact stereochemistry. Urinary serine:indoxyl sulfate and serine:hydroxyindole sulfate were found to be ratiometric biomarkers optimal for discriminating between pediatric IBD subtypes when using receiver operating characteristic (ROC) curves (Figure 2C) with excellent accuracy (AUC ~ 0.960–0.970, *p* < 3.00 × 10^−5^) as compared to indoxyl sulfate alone (AUC = 0.910, *p* = 1.60 × 10^−3^). Urine stability studies were also performed in this study (Supplementary Figure S5), which confirmed that indoxyl sulfate, hydroxyindoxyl sulfate and other urinary metabolites can tolerate delays to storage for up to 48 h even at room temperature without evidence of degradation/biotransformation. In contrast, certain urinary metabolites (e.g., serine, threonine, hypoxanthine, kynurenine) were stable for up to 48 h only if kept refrigerated (+4 °C) prior to freezing (−80 °C). As expected, the addition of sodium azide (1.0 mM) as a preservative to all urine samples upon collection did not alter metabolite responses as compared to untreated urine samples (*p* > 0.05).

### 2.4. Specific Urinary Biomarkers Following EEN or CS Therapy of Pediatric IBD Patients

**Figure 3:**
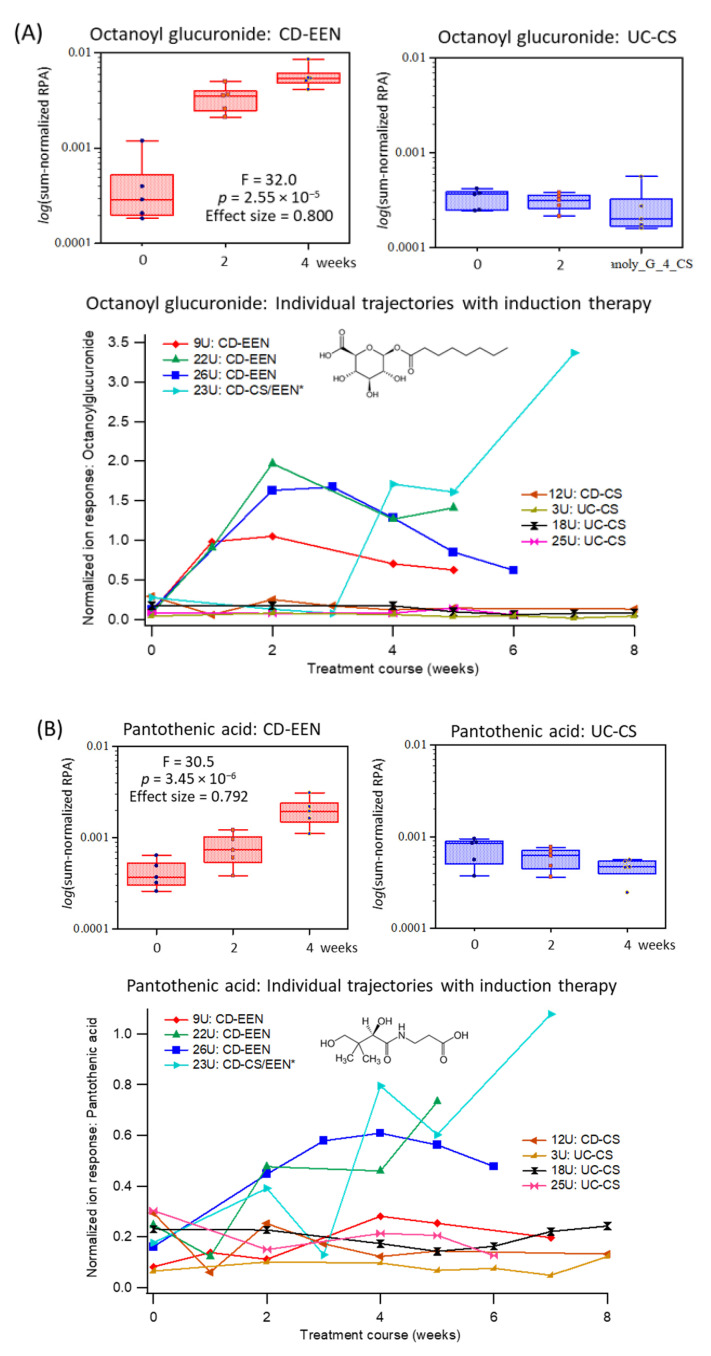
Top-ranked urinary biomarkers associated with adherence to EEN among pediatric IBD patients (*n* = 10), namely (**A**) octanoylglucuronide and (**B**) pantothenic acid. Box-whisker plots show a specific elevation in the excretion of both urinary metabolites following the initiation of EEN as compared to CS therapy at 2 and 4 weeks relative to baseline levels when using a repeat measures 2-way ANOVA with strong effect sizes. Additionally, metabolic trajectories for urinary octanoylglucuronide and pyridoxic acid are also shown for individual IBD patients in the two treatment arms (CD-EEN; UC/CD-CS, *n* = 8) over 8 weeks, including one CD patient who was later switched to EEN from CS after 2–3 weeks (CD-CS/EEN). This clinical treatment course change is more evident in the urinary excretion of octanoylglucuronide than pantothenic acid.

Clinical response, or clinical remission to induction therapy was assessed via protocol definitions (Supplementary Table S3). Two UC patients were non-responders to CS therapy following an 8-week oral prednisone treatment course. Repeat urine samples were collected from most patients, however, samples were less frequently provided at later time periods. A repeat measures 2-way ANOVA was initially performed on a subset of IBD cases (UC-EEN, *n* = 5; CD-CS, *n* = 5) at baseline with matching urine samples collected at 2 and 4 weeks following initiation of induction therapy. Table 3 demonstrates that eight urinary metabolites showed significant interaction effects (treatment × time) between EEN and CS treatment arms with moderate (0.40–0.60) to large effect sizes (>0.70). An unknown anion tentatively identified (level 3) as octanoyl glucuronide based on its diagnostic MS/MS spectrum (Supplementary Figure S6), as well as pantothenic acid and pyridoxic acid. The latter B vitamins are key constituents of the EEN formula, whereas octanoyl glucuronide is a biotransformed metabolite from the consumption of medium-chain triglycerides [26] prevalent in coconut or palm kernel oil (Supplementary Table S4). As expected, urinary octanoyl glucuronide is also strongly correlated to both pantothenic acid (*r* = 0.772, *n* = 97) and pyridoxic acid (*r* = 0.767, *n* = 97). These three urinary metabolites are excreted progressively higher in pediatric CD patients prescribed EEN, but remain low and unchanged in pediatric CD patients receiving CS therapy. Figure 3 depicts box plots for urinary octanoyl glucuronide and pantothenic acid that have the most striking temporal changes following EEN therapy despite analogous levels at baseline for both CD and UC children. Urinary metabolite trajectories are also shown for individual patients (*n* = 8) with samples collected for up to 8 weeks, including a CD patient who was initially prescribed CS, but later switched to EEN (after 3 weeks of therapy) due to an incorrect initial diagnosis. Other urinary biomarkers (Table 3) had less pronounced temporal changes when assessing their metabolic trajectories as compared to baseline and the CS treatment arm, such as pyridoxic acid and trigonelline (Supplementary Figure S7).

Dynamic changes in the urine metabolome in pediatric IBD patients following CS therapy also revealed a progressive decrease in the excretion of several endogenous urinary steroid conjugates as compared to baseline and the EEN treatment arm (Supplementary Figure S8), including cortolone glucuronide, hydroxyandrosterone glucuronide, tetrahydrocortisone glucuronide, and an unknown steroid anion (525.269:0.733:n, Supplementary Table S1). Nevertheless, mucosal healing likely takes place early during either CS or EEN treatment as reflected by a progressive lowering of serum CRP and FCP levels in pediatric IBD patients within 4 weeks of treatment initiation (Supplementary Figure S9). Supplementary Figure S10 depicts a correlation matrix among the 21 urinary metabolites having promising clinical utility to differentiate children with IBD in this study, as well as monitor treatment responses to EEN or CS therapy as compared to conventional inflammatory biomarkers (CRP, FCP). Overall, six distinct clusters of urinary metabolites are evident suggesting a common regulatory pathway and/or origin, including hydroxylated amino acids (serine, threonine), endogenous steroid glucuronides putatively modulated by oral prednisone, exogenous B vitamins or biotransformed nutrients from dietary intake of EEN, indole metabolites likely reflecting differences in gut microbiota composition, and energetic/oxidative stress metabolites (hypoxanthine, unknown cation: 222.078:0.849:p). Sialic acid was the only urinary metabolite (*p* < 0.05) correlated with FCP (*r* = 0.386) and serum CRP (*r* = 0.216), whereas urinary hypoxanthine was modestly associated only with FCP (*r* = 0.307).

**Table 2:** Top-ranked urinary biomarkers (sum-normalized) identified by MSI-CE-MS that differentiate pediatric CD (*n* = 18) from UC (*n* = 8) patients prior to induction therapy.

*m*/*z*:RMT:Mode	Metabolite ID	Median FC	*p*-Value ^1^	Effect Size ^1^
212.002:1.025:n	Indoxyl sulfate; HMDB0000682	2.03	0.00111	0.443
120.065:0.905:p	Threonine; HMDB0000167	0.51	0.00485	0.345
345.155:0.770:n	Unknown fatty acid; C_16_H_26_O_8_	1.93	0.00737	0.309
106.050:0.868:p	Serine; HMDB0000187	0.74	0.0122	0.273
308.099:0.791:n	Sialic acid; HMDB000230	1.89	0.0122	0.254
263.104:0.826:n	Phenylacetylglutamine; HMDB00006344	1.92	0.0144	0.261
137.046:1.039:p	Hypoxanthine; HMDB0000157	0.54	0.0144	0.178
222.080:0.849:p	5-(δ-Carboxybutyl)homocysteine ^2^	0.44	0.0198	0.239
209.092:0.887:p	Kynurenine; HMDB0000684	0.61	0.0268	0.204
227.997:0.979:n	5-Hydroxyindole sulfate ^2^	1.87	0.0357	0.210

^1^ Statistical significance determined by a Mann–Whitney U-test, *p* < 0.05 with effect size calculated using (Z^2^/N-1). ^2^ Putative identification (level 2) of unknown metabolites based on accurate mass, MS/MS, and mobility matching.

**Table 3:** A repeat measures 2-way ANOVA of sum-normalized log-transformed for monitoring changes in urine metabolome of pediatric IBD patients (*n* = 10) following EEN or CS induction therapy (0, 2, 4 weeks).

*m*/*z*:RMT:Mode	Metabolite ID	F-Test ^1^	*p*-Value ^1^	Effect Size ^1^
319.140:0.782:n	Octanoylglucuronide; ^2^ HMDB0010347	32.0	2.55 E-06	0.800
218.103:0.836:n	Pantothenic acid; HMBD0000210	30.5	3.45 E-06	0.792
182.046:0.948:n	Pyridoxic acid; HMDB0000017	10.4	0.00127	0.566
212.002:1.025:n	Indoxyl sulfate; HMDB0000682	6.46	0.00877	0.447
191.066:1.007:p	Unknown; C_6_H_10_N_2_O_5_	6.31	0.00952	0.441
138.055:0.909:p	Trigonelline; HMDB0000875	5.74	0.0132	0.418
201.113:1.218:n	Sebacic acid; HMDB0000792 ^2^	4.37	0.0307	0.353
308.078:1.302:n	Indoxyl glucuronide; ^2^ HMDB0010319	4.30	0.0320	0.350

^1^ Statistical significance (*p* < 0.05) based on interaction term (treatment × time) for *log*-transformed urine metabolome data with effect size calculated using partial eta^2^. ^2^ Putative identification (level 3) of unknown metabolites based on accurate mass, MS/MS, and mobility matching.

## 3. Discussion

Differentiation of CD from UC based on metabolic phenotyping has proven elusive, largely due to the clinical heterogeneity of adult IBD patients, and the confounding effects of long-term maintenance medications and surgeries on host metabolism and gut microbiome. We focused our study primarily on newly diagnosed pediatric IBD cases with active disease, with most children being treatment naïve (Table 1). To date, there have been few metabolomic studies evaluating urine as a noninvasive biomarker for diagnosis and monitoring of pediatric IBD. Previous studies have focused on comparisons to healthy controls while evaluating disease progression as related to weight loss and growth failure [27,28].

Perturbations in intestinal microbiota (i.e., dysbiosis) have long been implicated in IBD pathophysiology that directly modulate immune responses and nutritional homeostasis, where tryptophan metabolism plays a central role in microbiota–host crosstalk [29]. For example, indoxyl sulfate is primarily derived from dietary intake of tryptophan and subsequently metabolized into indole via tryptophanase by certain intestinal anaerobes prior to its absorption into circulation and subsequent primary (hydroxylation) and secondary (sulfation) metabolism in the liver. Urine metabolomic analyses were performed by MSI-CE-MS using a validated data workflow for authenticating a wide class of polar/hydrophilic metabolites (Supplementary Table S1; Figure 1) with stringent QC [23,24,25]. Sum or osmolality normalization of single-spot urine samples provided consistent outcomes for several urinary biomarkers associated with IBD subtypes and their treatment responses to induction therapy. This was needed to adjust for variations in hydration status since creatinine is not optimal for young children and adolescents having different muscle mass especially when undergoing drastic changes in habitual diet following EEN.

**Figure 4:**
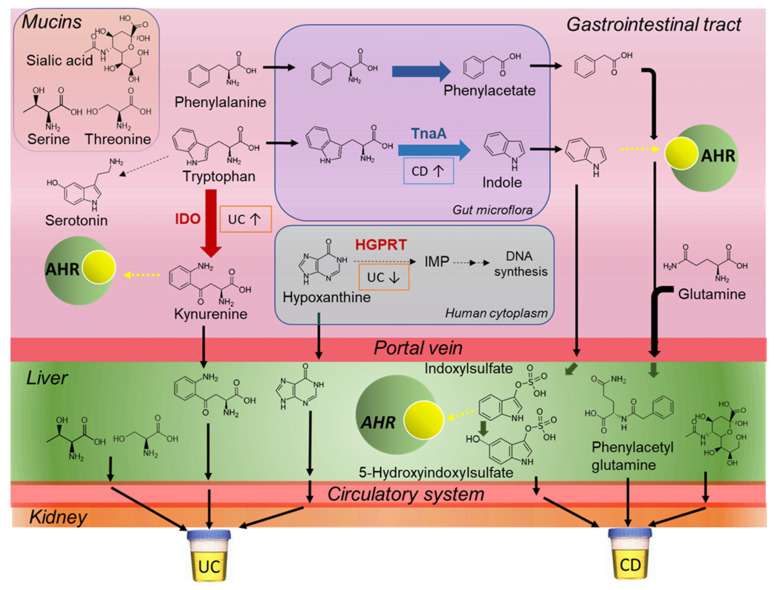
Proposed mechanisms that distinguish CD from UC pathophysiology based on differences in the urine metabolic phenotype of pediatric IBD subtypes prior to induction therapy. Key metabolic pathways include tryptophan/phenylalanine catabolism, mucin barrier function, and purine degradation pathways in the gut, liver and circulatory systems. Thick arrows represent enzymatic conversions and dotted arrow represent binding to AHR. AHR: aryl hydrocarbon receptor; IDO: indoleamine 2,3-dioxygenase; TnaA: tryptophanase, PAA: phenylacetic acid gene cluster; HGPRT: hypoxanthine-guanine phosphoribosyltransferase.

In this work, we discovered that children with CD have higher excretion of indoxyl sulfate and its oxidized metabolite, hydroxyindoxyl sulfate that was identified by MS/MS for the first time, along with corresponding lower kynurenine as compared to age-matched UC cases (Figure 2, Table 2). Elevated levels of indoxyl sulfate and other indole/aryl metabolites function as proinflammatory uremic toxins in circulation, which may contribute to epithelial barrier injury [30] in IBD patients with underlying gut dysbiosis. Similarly, phenylacetylglutamine also had higher excretion in patients with CD relative to UC. This has previously been reported to be associated with IBD disease course in children [27]. Furthermore, increased tryptophan catabolism via the kynurenine pathway is associated with greater endoscopic mucosal inflammation in UC patients [31]. We hypothesize (Figure 4) that higher urinary excretion of kynurenine may indicate an indoleamine 2,3-dioxygenase (IDO)-mediated dysregulation of host immune response as a key mechanism in early onset UC, whereas greater indoxyl sulfate, hydroxyindoxyl sulfate and phenylacetylglutamine elimination reflects a bacterial-mediated pathway that is more predominant in CD children. 

We also found differential excretion of two hydroxylated amino acids, namely serine and threonine in the urine of patients with UC as compared to CD (Figure 4). Threonine and serine are required for intestinal mucin synthesis to promote colonocyte and mucosal regeneration that is critical to barrier function [32,33]. Additionally, hypoxanthine was higher in the urine of UC as compared to CD patients. Disruptions to intestinal epithelial cell barrier function caused by energetic stress results in hypoxanthine loss during active inflammation [34]. Urinary hypoxanthine was also correlated to FCP suggesting that energetic/oxidative stress is more pronounced in UC as compared to colonic inflammation in CD (Figure 2 and Figure 4). Two unknown urinary metabolites also differentiate CD from UC children, which are tentatively identified by MS/MS as a dicarboxylic acid analog (345.155:0.777:n, C_16_H_26_O_8_; Supplementary Figure S4), and a sulfur amino acid conjugate (222.080:0.849:p, C_8_H_15_NO_4_S; Supplementary Figure S5) correlated to hypoxanthine (*r* = 0.566). Additionally, urinary sialic acid was found to have greater excretion in pediatric patients with CD versus UC, reflecting differences in IBD pathophysiology (Figure 2, Table 2), while also being correlated with FCP and serum CRP following induction therapy (Supplementary Figure S9). Greater sialic acid catabolism from colonic (glycated) mucin has been implicated in intestinal inflammation and colitis in murine models due to shifts in bacterial composition with increased sialidase activity that elicit a growth advantage for *E. coli* [35]. Overall, the ratio of serine:hydroxyindoxyl sulfate or serine:indoxylsulfate (Figure 2C) provide excellent accuracy (*AUC* ~ 0.965) for discriminating between the two major IBD subtypes in recently diagnosed children. However, further replication of these results is warranted in a larger and independent patient cohort. Stability studies also confirmed that IBD subtype-specific urinary metabolites can be reliably measured upon refrigeration for up to 2 days without the need for sodium azide as a preservative (Supplementary Figure S6). This feature offers practical utility in clinical settings, where children and families may provide samples that may have had delays in refrigeration or preservative addition, but still retain diagnostic performance.

Since metabolic profiles, especially that of urine, tend to be strongly influenced by short-term changes in diet [36], one of the challenges in this study was to distinguish metabolites/nutrients associated with intake of EEN formula from changes in mucosal healing and gut microbiota metabolism with clinical remission. As expected, initiation of EEN therapy constitutes a major change in normal oral feeding patterns in children from baseline as it is composed of a standardized liquid formula providing total caloric and nutritional requirements for an 8-week period [22]. In our study, 72% (*n* = 19) of recently diagnosed CD patients (including one UC patient) were prescribed EEN for the first time, and 68% (*n* = 13) achieved full remission or partial treatment response (32%, *n* = 6) after 8 weeks. Urinary octanoyl glucuronide was identified as the most specific biomarker for monitoring adherence to EEN (Table 3), increasing within 1 week of feeding with a median 15.5-fold increase at 4 weeks as compared to baseline (Figure 3A). Urinary octanoyl glucuronide is a major urinary metabolite of medium-chain triglycerides that is the major dietary fat in Peptamen^®^ 1.5 formula and other commercial EEN products. Metabolic trajectories for urinary octanoyl glucuronide also demonstrated excellent sensitivity and specificity to detect a delayed introduction of EEN in one patient (23U) after week 3 who was initially prescribed CS therapy due to an incorrect diagnosis. An earlier study reported that urinary excretion of octanoyl glucuronide peaks rapidly within 2 h after ingestion of medium-chain triglycerides by children, however, neither hexanoyl nor decanoyl glucuronides are detected [26]. Additionally, urinary pantothenic acid (vitamin B5) and pyridoxic acid (catabolite from pyridoxine or vitamin B6) were also found elevated in IBD patients receiving EEN as compared to their baseline measurements, as well as patients receiving CS (Figure 3, Supplementary Figure S7), with a strong interaction (treatment × time) effect size (Table 3). A recent multiomics study reported that IBD patients are prone to B vitamin deficiencies likely due to poor absorption and/or shorter bowel transit times as compared to healthy controls with lower pantothenic acid and nicotinic acid (vitamin B3) levels in circulation, but with corresponding higher amounts in stool [13]. In fact, urinary trigonelline, a methylated catabolite of niacin was also found modestly responsive to EEN therapy (Table 3), however, it was less significant than other B vitamins since it is enriched in commonly consumed foods (e.g., wheat flour) unlike pyridoxine and pantothenic acid. As a result, urinary metabolites from specific dietary fats or micronutrients prevalent in EEN formulations (Supplementary Table S4) allow for adherence monitoring of safe yet efficacious nutritional therapies in children with IBD, particularly in those patients who appear to be non-responders.

In contrast, there were less distinctive metabolic signatures in the urine of IBD patients following CS treatment since their habitual diet remained largely unchanged during the intervention period. As expected, pediatric IBD patients prescribed oral CS therapy while at home were evident based on their excretion of prednisone in urine as compared to baseline (Supplementary Table S1) that was not detected in the EEN treatment arm. Additionally, there were modest decreases in the excretion of several endogenous steroids after 4 weeks of CS therapy relative to their baseline and the EEN treatment arm (Supplementary Figure S8), tentatively identified as major cortisol metabolites, such as cortolone and tetrahydrocortisone as their glucuronide conjugates. This result coincides with lower CS-mediated inflammation in treated pediatric UC patients that is distinct from urine metabolic phenotype changes in CD cases following EEN; however, long-term CS therapy is not recommended in children due to adverse effects on normal growth and development. In general, both induction therapies were able to induce remission in most pediatric IBD patients. However, two children with UC did not respond to CS therapy; this was not observed in patients who received EEN, which is recommended for all newly diagnosed children with CD [5].

## 4. Materials and Methods

### 4.1. Pediatric IBD Study Cohort

This study was approved by the Hamilton Integrated Research Ethics Board (#15-365) and parental consent was obtained for all the participants. The study enrolled children from 5 to 18 years old who had been diagnosed with IBD by endoscopy, histology and radiography at McMaster Children’s Hospital. Patients were included if they were admitted to hospital to be initiated on EEN therapy using Peptamen^®^ 1.5 (Nestlé Health Sciences, Bridgewater, NJ, USA) at 120% of their daily caloric needs, or intravenous CS (1 mg/kg/day methylprednisolone) for induction of IBD remission [22]. Patients were discharged home once full volumes of EEN feeds were achieved (<72 h upon admission), and other than formula, they were allowed to consume only clear fluids orally. Patients on CS therapy were discharged home once symptoms improved in the hospital, when they were transitioned to oral prednisolone while maintaining their normal diet throughout the intervention. Patients were excluded if they were younger than 5 years, received antibiotic therapy, or did not require admission to hospital. None of the patients have undergone intestinal resection surgery. Clinical improvement was defined as a decrease in the pediatric Crohn’s disease activity index (PCDAI) or modified pediatric ulcerative colitis activity index (PUCAI) from baseline enrolment, and clinical remission was defined as a score of ≤ 10 in each scale [22]. Active disease was defined as all baseline clinical, and biochemical (CRP, albumin, hemoglobin, FCP) parameters. Remission was defined as clinical remission and biochemical markers within normal limits (e.g., CRP ≤ 1 mg/L; FCP ≤ 250 μg/g). Response to treatment, but not clinical remission was defined as improvement in disease activity scores, and biochemical parameters from active disease, whereas no response was defined as a worsening of disease symptoms from baseline. Further details on this longitudinal study to evaluate of the efficacy of EEN and CS treatment on pediatric IBD patients are described elsewhere [22].

### 4.2. Urine Sample Collection, Storage, and Workup Procedure

All urine samples included in this study were collected prior to and following induction therapy (i.e., EEN or CS) at McMaster Children’s Hospital. Single-spot urine samples were collected randomly in the morning. Following collection, 1.0 mM of sodium azide was added to all urine samples as an antimicrobial preservative and then samples were stored in a fridge before being transferred to a freezer at −80 °C. All urine samples (25 μL aliquot) were thawed slowly on ice and then diluted (from 5 to 10-fold) in ultra-grade LC-MS water containing two internal standards, 3-chloro-l-tyrosine (Cl-Tyr, 10 µM) and sodium 2-naphthalenesulfonate (NMS, 10 µM), which was followed by mixing using a vortex for 30 s. Pooled quality control (QC) samples were prepared from a random subset of all urine samples (*n* = 30) for assessment of technical precision and long-term instrumental signal drift. Urine samples were collected at baseline (*n* = 28) and following treatment interventions (*n* = 67) from pediatric CD and UC patients over an 8-week period; however, urine samples were not provided consistently at every time point by all participants, and one patient later dropped out of the clinical trial. Osmolality was measured using Advanced Micro-Osmometer 3300 (Fisher Scientific Company, Waltham, MA, USA), whereas urinary creatinine concentrations were quantified by MSI-CE-MS that was linearly correlated to urine osmolality [25].

### 4.3. Urinary Metabolite Stability Studies

Five random single-spot urine samples were collected from five healthy volunteers for metabolite stability studies. Each sample was placed on ice and a pooled sample was prepared within 1 h upon initial urine collection. Aliquots of this pooled urine sample were stored at either room temperature (~22 °C) or in a fridge (4 °C) for 6, 12, 24, 36, and 48 h. After assigned storage duration/temperature, 1.0 mM sodium azide was then added and samples were then stored at −80 °C with each sample performed in triplicate. Six urine aliquots of a pooled urine sample were also prepared and immediately transferred to a freezer at −80 °C with or without 1.0 mM sodium azide as controls. The same dilution and analysis protocol was performed when using MSI-CE-MS as described for pediatric IBD urine samples.

### 4.4. Nontargeted Metabolic Phenotyping of Urine by MSI-CE-MS

Urine metabolomic analyses were performed on an Agilent G7100A CE system (Agilent Technologies Inc., Mississauga, ON, Canada) equipped with a coaxial sheath liquid Jetstream electrospray ion source with heated nitrogen gas to an Agilent 6550 iFunnel Q-TOF-MS system. Separations were performed using an uncoated fused silica capillary (Polymicro Technologies, Phoenix, AZ, USA) with an inner diameter of 50 µm, outer diameter of 360 µm, and total length of 110 cm with a voltage of 30 kV at 25 °C. Each diluted urine sample was analyzed by MSI-CE-MS under two conditions based on a background electrolyte (BGE) composed of 1.0 M formic acid with 15% vol acetonitrile (pH = 1.80) and 50 mM ammonium bicarbonate (pH = 8.50) when using positive (i.e., basic/zwitter-ionic metabolites) and negative (i.e., acidic metabolites) ion mode detection, respectively [23,24,25]. The sheath liquid composition for electrospray formation when using the coaxial sheath liquid interface in MSI-CE-MS was composed of 60% vol methanol with 0.1% vol formic acid under positive ion mode detection, and 50% vol MeOH under negative ion mode detection with full-scan data acquisition over a mass range of *m*/*z* 50–1700. Prior to sample injection, the capillary was conditioned with BGE for 15 min to ensure adequate equilibration. A seven sample serial injection format was used in MSI-CE-MS for multiplexed separations of urine samples, which utilized an alternating hydrodynamic injection sequence of 5 s (at 100 mbar) for each sample followed by 40 s (at 100 mbar) of BGE that served as spacer plug between each pair of diluted urine sample [25]. Briefly, all urine samples (*n* = 95) collected from a longitudinal study of pediatric IBD patients in both EEN and CS treatment arms were fully randomized for analysis as a single batch. Three urine samples were introduced in duplicate in each run by MSI-CE-MS, where sample pairs are diluted using a distinctive injection pattern (i.e., 1:1, 1:2 or 2:1) to facilitate identification of exact sample position via temporal signal pattern recognition [23,24,25]. Additionally, a QC (i.e., pooled urine) as a seventh sample was introduced in a random position in every run, which enables robust correction of interbatch effects as required for longitudinal and/or large-scale metabolomic studies (23). The ESI conditions were Vcap = 3500 V, nozzle voltage = 2000 V, nebulizer gas = 8 psi, sheath gas = 3.5 L/min at 200 °C, and drying gas = 16 L/min at 200 °C for both ionization modes. Additionally, the MS voltage settings were fragmentor = 120 V, skimmer = 65 V and Oct1 RF = 750 V. Structural elucidation of unknown urinary metabolites was performed by collisional-induced dissociation when using auto MS/MS and targeted MS/MS modes on a QTOF-MS system with collision energies at 10, 20 or 30 V and 40 V. A combination of deposited MS/MS spectral databases (e.g., HMDB), in-silico fragmentation (e.g., MetFrag), and manual annotation was used for MS/MS spectral interpretation previously applied to identify urinary biomarkers of irritable bowel syndrome [25].

### 4.5. Metabolomics Data Processing and Statistical Analysis

Raw data (.d format) was processed using Mass Hunter Workstation Software (Qualitative Analysis, version B.6.00, Agilent Technologies, Santa Clara, CA, USA, 2012). Initial molecular feature detection and metabolite identification was performed using Mass Hunter Molecular Feature Extractor, Molecular Formula Generator tools and an in-house compound database [25]. Molecular features were extracted using a 10 ppm mass window and ions were annotated by their accurate mass (*m*/*z*), relative migration time (RMT) as compared to an internal standard (Cl-Tyr or NMS), and ionization mode (m) used for detection (p: positive; n: negative). Peak smoothing was performed using a quadratic/cubic Savitzky–Golay function (15 points) prior to peak integration. Peak areas and migration times for all molecular features and internal standards were transferred to Excel (Microsoft Office, Version 2102) and saved as .csv file. Next, determination of relative peak area (RPA) and RMT, and coefficient of variation (CV) from QC samples in every run was calculated, where urinary metabolites with high technical variance (CV > 35%), low detection frequency (< 75%) or exogenous compounds from drug administration were excluded from the final data matrix [25]. Subsequently, ion responses for authentic urinary metabolites consistently measured in most urine samples were normalized to osmolality or preferably normalized to the sum of all urinary metabolite responses by MSI-CE-MS to adjust for differences in hydration status. Multivariate data analysis was performed using Metaboanalyst 5.0 [37], including principal component analysis (PCA), partial least-squares-discriminant analysis (PLS-DA), and receiver operating characteristic (ROC) curves. In all cases, missing values were replaced with half of the lowest detected value, whereas metabolomic data sets were (generalized) *log* transformed and autoscaled unless otherwise stated. Additionally, data normality and effect size calculations were performed using the Statistical Package for the Social Science (SPSS, version 21). Normality assumption was violated for majority (73%) of urinary metabolites based on Shapiro–Wilk test (α = 0.05). As a result, nonparametric univariate test (Mann–Whitney *U*-test) was performed on the sum normalized urine metabolomic data matrix for differentiation of IBD subtypes at baseline. A repeat measures 2-way analysis of variance (ANOVA, interaction effect, treatment × time) on log-transformed and sum normalized data sets was performed to identify urinary biomarkers associated with EEN as compared to CS induction therapy using matching/repeat urine samples collected after 2 and 4 weeks from baseline for each IBD patient. Urinary metabolic trajectories were also plotted for individual IBD patients over the 8-week intervention period for EEN and CS treatment arms.

## 5. Conclusions

In summary, urine metabolic phenotyping of pediatric IBD identified several discriminating metabolites that may provide a simple and noninvasive way to differentiate pediatric CD from UC, as required for optimal disease management. Additional urinary metabolites may also serve as promising biomarkers for monitoring treatment adherence and clinical response, particularly in patients receiving EEN induction therapy. Importantly as well, urinary biomarkers were found to be adequately stable with refrigeration without need for preservatives. A major limitation in this single-center retrospective pilot study was the modest and unbalanced sample size without a healthy control group. Nonetheless, a rigorous metabolomics data workflow was applied to reduce false discoveries. Future work will validate these findings in a larger cohort of recently diagnosed children with IBD and other chronic intestinal conditions. Further elucidation of the exact chemical structures of certain unknown IBD-specific metabolites in urine will also be performed. Stool metabolomic analyses may provide deeper insights into the underlying mechanisms of EEN for alleviating colonic inflammation attributed to intestinal dysbiosis, which are distinct from the immunosuppressive and anti-inflammatory effects of CS therapy.

## Figures and Tables

**Table 1:** Pediatric patients diagnosed with inflammatory bowel disease (IBD) with clinical measurements based on mean values and errors as ±1 SD. All biochemical measurements were derived from serum, urine or stool, whereas disease location was based on endoscopy and magnetic resonance enterography imaging.

Criteria	CD (*n* = 18)	UC (*n* = 8)
Age	13 ± 2	12 ± 3
Sex; male:female	11:7	4:4
Diagnosis < 1 month (%)	13 (72%)	6 (75%)
EEN; CS treatment arm (n)	15; 3	1; 7
Serum CRP (mg/L) ^1^	40 ± 40	36 ± 65
FCP (µg/g) ^1^	3240 ± 2210	2558 ± 1150
Hemoglobin (g/L) ^1^	109 ± 18	110 ± 16
ESR (mm/h) ^1^	37 ± 26	43 ± 23
Albumin (g/L) ^1^	28.2 ± 4.8	30.4 ± 2.3
WBC (×10^9^/L) ^1^	8.1 ± 2.3	7.3 ± 2.0
Urinary creatinine (mg/L)	11.0 ± 5.2	9.1 ± 6.6
Urine osmolality (mOsm/kg)	480 ± 190	408 ± 250
*Disease location:*		
Ileocolonic	11	NA
Ileocolonic + UGI	2	NA
Colonic	2	8
Colonic + UGI	3	NA
*Maintenance medications:* ^2^		
Biologic	1 (2)	0 (0)
Immunomodulator	2 (10)	0 (0)
5-ASA	0 (2)	1 (5)
Biologic + Immunomodulator	2 (2)	1 (2)
*Clinical outcomes:* ^3^		
Remission; Response; No response	11; 7; 0	4; 1; 2

^1^ There were no significant differences (*p* > 0.05, Mann–Whitney U test) measured in serological and stool biomarkers of inflammation between CD and UC children. ^2^ Maintenance medications prior to and (after) EEN or CS therapy, including biologics: adalimumab or infliximab; immunomodulators: methotrexate or azathioprine; 5-ASA: 5-aminosalicylic acid (mesalamine); combination biologics: adalimumab + azathioprine or methotrexate. ^3^ Clinical improvement was defined as a decrease in the PUCAI or modified PCDAI from baseline enrolment, and clinical remission was defined as a score of <10 in each scale. Remission was defined as clinical remission and biochemical markers within normal limits (FCP < 250 μg/g). Abbreviations include, CD: Crohn’s disease; ESR: erythrocyte sedimentation rate; FCP: fecal calprotectin; UC: ulcerative colitis; PCDAI: pediatric Crohn’s disease activity index; PUCAI: pediatric ulcerative colitis activity index; UGI: upper gastrointestinal tract. One UC patient dropped out during the clinical intervention study.

## Data Availability

All urine metabolomics data used in this work is available as a Supplementary excel file from deidentified pediatric IBD patients.

## Supplementary Materials

The following are available online at https://www.mdpi.com/article/10.3390/metabo11040245/s1, Figure S1: Characterization of an unknown anion in urine identified (level 2) as propofol glucuronide by MS/MS, Figure S2: Characterization of an unknown anion in urine identified (level 2) as phenylsulfate by MS/MS, Figure S3: Putative identification of an unknown organic acid anion in urine (level 3) elevated in CD as compared to UC children by MS/MS, Figure S4: Putative identification of an unknown cationic/thiol derived metabolite in urine (level 3) that was elevated in CD as compared to UC children by MS/MS, Figure S5: Representative plots illustrating the chemical stability of select urinary metabolites, Figure S6: Putative identification of an unknown anion in urine (level 3) that was elevated following EEN as compared to CS therapy, Figure S7: Secondary urinary biomarkers associated with adherence to EEN in pediatric IBD patients, namely pyridoxic acid and trigonelline, Figure S8: Supervised multivariate data analysis of the urine metabolome from pediatric IBD patients after 4 weeks of EEN or CS therapy, Figure S9: The efficacy of complementary induction therapy treatments to reduce active inflammation in pediatric IBD patients based on assessment of serum CRP and FCP, Figure S10: Correlation matrix on 21 urinary biomarkers associated with differentiation of pediatric IBD subtypes and treatment responses to EEN therapy identified in this work, Table S1: Summary of 132 urinary metabolites detected in urine samples of pediatric IBD patients, Table S2: Top-ranked ratiometric biomarkers identified by MSI-CE-MS that differentiate pediatric CD from UC in osmolality-normalized urine, Table S3: Classification criteria of inflammatory state for each sample based on disease score and inflammatory markers, Table S4: Constituents of EEN formula based on information provided by the manufacturer, Excel data file: Urine Metabolome Data File_Pediatric IBD.xlsx.
