# Supplementary material for: Urinary Metabolites Enable Differential Diagnosis and Therapeutic Monitoring of Pediatric Inflammatory Bowel Disease
Source: Metabolites. 2021 Apr 15;11(4):245. doi: 10.3390/metabo11040245 (PMC8071482; doi:10.3390/metabo11040245)
Supplement: Supplementary file 1 [file metabolites-11-00245-s001.zip › Supplemental Files/IBD_Britz-SI.docx]

**Supplemental Material**

Urinary Metabolites Enable Differential Diagnosis and Therapeutic Monitoring of Pediatric Inflammatory Bowel Disease

Mai Yamamoto,^1^ Meera Shanmuganathan,^1^ Lara Hart,^2^ Nikhil Pai^2^, and Philip Britz-McKibbin^1*^

*^1^ Department of Chemistry and Chemical Biology, McMaster University, Hamilton, ON, Canada*

*^2^ Department of Pediatrics, Division of Pediatric Gastroenterology, McMaster Children’s Hospital, McMaster University, Hamilton, ON, Canada*

**Correspondence:** Philip Britz-McKibbin, Department of Chemistry and Chemical Biology, McMaster University, Hamilton, ON, L8S 4M1, Canada

**E-mail:** britz@mcmaster.ca

**Fax:** +1-905-522-2509

**Table S1- S4; Figure S1-S10**

**Table S1.** Summary of 132 urinary metabolites detected in urine samples of pediatric IBD patients with *m/z*, RMT, ionization mode (p = positive, n = negative), most likely molecular formula, chemical or putative chemical name, and confidence level of structural identification following search on HMDB.

| *m/z:*RMT:mode | Putative ID | Molecular Formula | Metabolite  Class | Confirmed  Level^b^ |
| --- | --- | --- | --- | --- |
| 87.0452:1.607:n | Butyric acid | C4H8O2 | organic acid | 1 |
| 89.0244:1.131:n | Lactic acid | C3H6O3 | organic acid | 1 |
| 117.0193:1.826:n | Succinic acid | C4H6O4 | organic acid | 1 |
| 128.0353:1.102:n | Oxo-proline | C5H7NO3 | amino acid | 1 |
| 131.035:1.609:n | Glutaric acid | C5H8O4 | organic acid | 2 |
| 132.0302:1.025:n | Aspartic acid | C4H7NO4 | amino acid | 1 |
| 135.0299:0.995:n | Threonic acid | C4H8O5 | organic acid | 1 |
| 145.0506:1.473:n | 2-Methylglutaric acid | C6H10O4 | organic acid | 2 |
| 156.0657:0.917:n | Tiglylglycine | C7H11NO3 | organonitrogen | 3 |
| 157.051:1.429:n | Isopropylmaleate | C7H10O4 | organic acid | 2 |
| 159.1027:0.861:n | 7-Hydroxyoctanoic acid | C8H16O3 | organic acid | 3 |
| 160.0615:0.911:n | Aminoadipic acid | C6H11NO4 | amino acid | 1 |
| 166.0146:1.014:n | Quinolinic acid | C7H5NO4 | organic acid | 3 |
| 167.0201:0.968:n | Uric acid | C5H4N4O3 | organic acid | 1 |
| 172.9912:1.135:n | Phenyl sulfate | C6H6O4S | phenylsulfate | 2 |
| 178.051:0.914:n | Hippuric acid | C9H9NO3 | organic acid | 1 |
| 181.0506:0.903:n | 3-(3-Hydroxyphenyl)-3-hydroxypropanoic acid (HPHPA) | C9H10O4 | organic acid | 3 |
| 182.0459:0.948:n | 4-Pyridoxic acid | C8H9NO4 | organic acid | 1 |
| 184.0977:0.876:n | 2-Hepteneoylglycine | C9H15NO3 | amino acid | 3 |
| 187.0071:1.059:n | *p*-Cresol sulfate | C7H8O4S | phenylsulfate | 1 |
| 188.0353:0.926:n | Kynurenic acid | C10H7NO3 | Quinoline | 1 |
| 188.0558:1.35:n | Acetylglutamate | C7H11NO5 | amino acid | 3 |
| 191.0552:0.895:n | Quinic acid | C7H12O6 | organic acid | 1 |
| 193.0357:0.882:n | Glucuronic acid | C6H10O7 | organic acid | 1 |
| 195.0524:0.888:n | Dimethyluric acid/gluconate | C7H8N4O3 | purine | 3 |
| 197.0455:0.900:n | *Unknown* | C5H6N6O3 | NA | 4 |
| 201.1129:1.218:n | Sebacic acid | C10H18O4 | organic acid | 3 |
| 212.0023:1.025:n | Indoxyl sulfate | C8H7NO4S | phenylsulfate | 1 |
| 218.1034:0.836:n | Pantothenic acid | C9H17NO5 | organonitrogen | 1 |
| 222.9916:0.973:n | *Unknown* | C9H4O7 | NA | 4 |
| 225.0629:0.860:n | 5-Acetylamino-6-formylamino-3-methyluracil | C8H10N4O4 | NA | 3 |
| 227.9968:0.979:n | 5-Hydroxy-6-indolyl-O-sulfate | C8H7NO5S | phenylsulfate | 2 |
| 241.1193:0.824:n | *Unknown* | C7H14N8O2 | NA | 4 |
| 243.0771:0.845:n | Indolylacryloylglycine | C13H12N2O3 | amino acid | 3 |
| 263.1037:0.826:n | Phenylacetyl glutamine | C13H16N2O4 | amino acid | 1 |
| 269.150:0.805:n | *Unknown* | C8H22N4O6 | NA | 4 |
| 283.0823:0.809:n | *p*-Cresol glucuronide | C13H16O7 | phenolic glycoside | 3 |
| 287.0227:0.912:n | Dihydroxyphenyl-γ-valerolactone sulfate | C11H12O7S | phenylsulfate | 3 |
| 290.0882:0.796:n | 2,3-Dehydro-2-deoxy-N-acetylneuraminic acid | C11H17NO8 | amino sugar | 1 |
| 302.114:0.812:n | Indoleacetyl glutamine | C15H17N3O4 | amino acid | 2 |
| 308.0776:1.302:n | Indoxyl glucuronide | C14H15NO7 | O-glucuronide | 3 |
| 308.0987:0.791:n | *N*-Acetylneuraminic acid | C11H19NO9 | amino sugar | 1 |
| 319.140:0.782:n | Octanoyl glucuronide | C14H24O8 | O-glucuronide | 3 |
| 331.1757:0.777:n | Neomenthol glucuronide | C16H28O7 | O-glucuronide | 4 |
| 336.0725:0.794:n | Indole-3-carboxylic acid glucuronide | C15H15NO8 | O-glucuronide | 3 |
| 338.0881:0.791:n | Hydroxymethoxyindole glucuronide | C15H17NO8 | O-glucuronide | 3 |
| 345.1553:0.770:n | *Unknown* | C16H26O8 | NA | 3 |
| 347.0853:0.826:n | *Unknown* | C23H12N2O2 | NA | 4 |
| 350.088:0.788:n | Indole-3-acetic-acid-O-glucuronide | C16H17NO8 | O-glucuronide | 3 |
| 352.0868:0.863:n | *Unknown* | C16H19NO6S | NA | 3 |
| 407.2803:0.750:n | Cholic acid | C24H40O5 | steroid | 1 |
| 464.3018:0.737:n | Glycocholic acid | C26H43NO6 | steroid | 1 |
| 481.2439:0.742:n | Hydroxyandrosterone-3-glucuronide | C25H38O9 | steroid | 3 |
| 525.2688:0.733:n | *Unknown* | C21H36N9O7 | NA | 4 |
| 539.2493:0.733:n | Tetrahydrocortisone-glucuronide | C27H40O11 | steroid | 3 |
| 541.2649:0.729:n | Cortolone-glucuronide | C27H42O11 | steroid | 2 |
| 543.2811:0.725:n | Cortol-3-glucuronide | C27H44O11 | steroid | 2 |
| 632.2044:0.717:n | Sialyllactose | C23H39NO19 | amino sugar | 1 |
| 673.2309:0.713:n | Sialyl-*N*-acetyllactosamine | C25H42N2O19 | amino sugar | 1 |
| 62.06:0.577:p | Ethanolamine | C2H7NO | amine | 1 |
| 76.0393:0.743:p | Glycine | C2H5NO2 | amino acid | 1 |
| 76.0757:0.594:p | Trimethylamine-*N*-oxide | C3H9NO | organonitrogen | 1 |
| 90.055:0.795:p | Alanine | C3H7NO2 | amino acid | 1 |
| 104.0706:0.94:p | Dimethylglycine | C4H9NO2 | amino acid | 1 |
| 104.0706:0.702:p | γ-Aminobutyric acid (GABA) | C4H9NO2 | amino acid | 1 |
| 104.1069:0.618:p | Choline | C5H14NO | organonitrogen | 1 |
| 106.0499:0.868:p | Serine | C3H7NO3 | amino acid | 1 |
| 114.0662:0.656:p | Creatinine | C4H7N3O | amino acid | 1 |
| 118.0611:0.737:p | *Unknown* | C3H7N3O2 | NA | 4 |
| 120.0652:0.905:p | Threonine | C4H9NO3 | amino acid | 1 |
| 129.0659:0.774:p | Dihydrothymine | C5H8N2O2 | purine | 3 |
| 131.1179:0.752:p | Acetylputriscine | C6H14N2O | amine | 1 |
| 132.0768:0.782:p | Creatine | C4H9N3O2 | amino acid | 1 |
| 133.0969:0.628:p | Ornithine | C5H12N2O2 | amino acid | 1 |
| 137.0457:1.039:p | Hypoxanthine | C5H4N4O | purine | 1 |
| 138.055:0.909:p | Trigonelline | C7H7NO2 | organic acid | 1 |
| 141.0659:0.734:p | Imidazole propionate | C6H8N2O2 | organic acid | 2 |
| 146.0924:0.742:p | 4-Guanidinobutanoate | C5H11N3O2 | organic acid | 3 |
| 147.0764:0.926:p | Glutamine | C5H10N2O3 | amino acid | 1 |
| 147.1128:0.631:p | Lysine | C6H14N2O2 | amino acid | 1 |
| 150.0583:0.915:p | Methionine | C5H11NO2S | amino acid | 1 |
| 156.0767:0.667:p | Histidine | C6H9N3O2 | amino acid | 1 |
| 162.1125:0.757:p | Carnitine | C7H15NO3 | organonitrogen | 1 |
| 163.1077:0.654:p | 5-Hydroxylysine | C6H14N2O3 | amino acid | 1 |
| 164.0748:0.769:p | Propyl-*S*-cysteine | C6H13NO2S | amino acid | 3 |
| 166.0723:0.744:p | Methylguanine | C6H7N5O | purine | 1 |
| 166.0863:0.94:p | Phenylalanine | C9H11NO2 | amino acid | 1 |
| 170.0924:0.681:p | 3-Methylhistidine | C7H11N3O2 | amino acid | 1 |
| 176.0658:0.876:p | Acetyl-aspartic acid | C6H9NO5 | amino acid | 3 |
| 182.0809:0.964:p | Tyrosine | C9H11NO3 | amino acid | 1 |
| 189.1598:0.651:p | Trimethyllysine | C9H20N2O2 | amino acid | 1 |
| 190.1191:0.954:p | Homocitrulline | C7H15N3O3 | amino acid | 1 |
| 191.0661:1.007:p | Aspartyl-glycine | C6H10N2O5 | amino acid | 3 |
| 195.0764:0.895:p | Aminohippuric acid | C9H10N2O3 | organic acid | 3 |
| 204.123:0.796:p | Acetyl-carnitine | C9H17NO4 | organonitrogen | 1 |
| 205.0972:0.938:p | Tryptophan | C11H12N2O2 | amino acid | 1 |
| 209.0921:0.887:p | Kynurenine | C10H12N2O3 | amino acid | 1 |
| 217.1294:0.869:p | Acetyl-arginine | C8H16N4O3 | amino acid | 3 |
| 222.0796:0.849:p | 5-(delta-carboxybutyl) Homocysteine | C8H15NO4S | amino acid | 2 |
| 223.0747:0.866:p | Cystathionine | C7H14N2O4S | amino acid | 1 |
| 232.1543:0.829:p | Butyryl carnitine | C11H21NO4 | organonitrogen | 2 |
| 238.0916:1.064:p | Xylosylserine | C8H15NO7 | amino sugar | 2 |
| 241.0311:0.946:p | Cystine | C6H12N2O4S2 | amino acid | 1 |
| 243.0981:0.91:p | Thymidine | C10H14N2O5 | pyrimidine | 2 |
| 244.1543:0.849:p | Tiglylcarnitine | C12H21NO4 | organonitrogen | 3 |
| 258.1084:0.862:p | Methylcytidine | C10H15N3O5 | pyrimidine | 3 |
| 259.0918:0.893:p | Ribothymidine | C10H14N2O6 | pyrimidine | 2 |
| 269.1238:0.926:p | Acetylcarnosine | C11H16N4O4 | amino acid | 1 |
| 276.1442:0.883:p | Glutaryl-carnitine | C12H21NO6 | organonitrogen | 3 |
| 282.1197:0.872:p | Methyl adenosine | C11H15N5O4 | purine | 1 |
| 286.2013:0.886:p | Fumaric acid, 2-dimethylaminoethyl heptyl ester | C18H25N2O | NA | 3 |
| 290.1598:0.897:p | Methylglutarylcarnitine | C13H23NO6 | organonitrogen | 3 |
| 291.1305:0.827:p | Arginosuccinate | C10H18N4O6 | amino acid | 3 |
| 298.097:0.656:p | Methylthioadenosine | C11H15N5O3S | purine | 2 |
| 298.1146:1.058:p | 1- or 2- or 3'-O-Methylguanosine | C11H15N5O5 | purine | 2 |
| 304.1755:0.908:p | Pimelyl carnitine | C14H25NO6 | organonitrogen | 3 |
| 304.2109:0.92:p | Hydroxyoctanoyl carnitine | C15H29NO5 | organonitrogen | 3 |
| 312.1297:1.039:p | Dimethyl guanosine | C12H17N5O5 | purine | 1 |
| 325.165:0.777:p | Galactosyl-hydroxylysine | C12H24N2O8 | amino acid | 1 |
| 367.15:1.065:p | Mannopyranosyl-Trptophan | C17H22N2O7 | amino sugar | 1 |
| 399.1451:0.638:p | *S*-Adenosylmethionine | C15H23N6O5S | amino acid | 3 |
| 487.2117:0.854:p | Glucosylgalactosyl-hydroxylysine | C18H34N2O13 | amino acid | 1 |
| 194.0458:0.921:n | Salicyluric acid ^a^ | C9H9NO4 | organic acid/ drug metabolite | 1 |
| 204.0666:0.876:n | Indole lactic acid ^a^ | C11H11NO3 | organic acid | 1 |
| 230.0127:0.933:n | Paracetamol sulfate ^a^ | C8H9NO5S | sulfar conjugate | 1 |
| 263.629:0.953:n  [M-2H]^2-^ | Unknown bile acid glycine sulfate conjugate adduct ^a^ | C26H43NO8S | steroid | 3 |
| 353.1597:0.766:n | Propofol glucuronide ^a^ | C18H26O7 | drug metabolite | 2 |
| 359.1857:0.587:n | Prednisolone ^a^ | C21H28O5 | drug metabolite | 2 |
| 369.1545:0.760:n | Hydroxypropofol glucuronide ^a^ | C18H26O8 | drug metabolite | 2 |
| 154.0499:0.887:p | Mesalamine ^a^ | C7H7NO3 | drug metabolite | 2 |
| 262.1028:1.079:p | Asp-Gln ^a^ | C9H15N3O6 | amino acid | 2 |
| 288.217:0.941:p | Octanoylcarnitine ^a^ | C15H29NO4 | organonitrogen | 1 |

*^a^ Compounds that were missing in more than 50% of total samples in this study. ^b^ Confidence level for urinary metabolite identification ranged from 1 = confirmed by spiking with standard, 2 = likely identified based on accurate mass, consistent mobility and MS/MS, 3 = putative identification based on accurate mass and consistent mobility, and 4 = unknown chemical structure with no likely candidate from HMDB search.*

**Table S2.** Top-ranked ratiometric biomarkers identified by MSI-CE-MS that differentiate pediatric CD from UC in osmolality-normalized urine at baseline prior to therapeutic intervention.

| Metabolite | HMDB ID | FC | *p*-value | Effect size |
| --- | --- | --- | --- | --- |
| Indoxyl sulfate | 0000682 | 2.02 | 0.00127 | 0.415 |
| Sialic acid (*N*-Acetylneuraminic acid) | 0000230 | 2.23 | 0.00510 | 0.357 |
| 5-Hydroxyindoxy sulfate | -- | 1.94 | 0.00627 | 0.325 |
| Kynurenine | 0000684 | 0.52 | 0.00903 | 0.273 |
| Threonine | 0000167 | 0.49 | 0.0143 | 0.240 |
| 345.155:0.770:n; C_16_H_26_O_8_ | *Unknown* | 2.91 | 0.0145 | 0.239 |
| Serine | 0000187 | 0.72 | 0.0196 | 0.218 |
| 222.080:0.849:p; C_8_H_15_NO_4_S | *Unknown* | 0.38 | 0.0263 | 0.198 |
| Hypoxanthine | 0000157 | 0.70 | 0.0303 | 0.188 |
| Phenylacetylglutamine |  | 1.49 | 0.0589 | 0.143 |

** Statistical significance calculated by a Mann-Whitney U-test, p < 0.05; Effect size is calculated using (Z^2^/N-1), where FC = median fold-change*

**Table S3.** Classification criteria of inflammatory state for each sample based on disease score (PCDAI/PUCAI) and inflammatory markers.

| Classification | Criteria |
| --- | --- |
| Active flaring/inflammation | FCP > 250 μg/g or CRP > 1.0 mg/L |
| Healing | FCP decreased from baseline or CRP decreased, OR disease score decreased from baseline |
| Remission | FCP ≤ 250 μg/g or CRP ≤ 1.0 mg/L |

*FCP: Fecal calprotectin; CRP: C-reactive protein*

**Table S4.** Constituents of EEN formula (Peptamen® 1.5) based on information provided by the manufacturer (Nestlé Health Science, www.nestlenutritionstore.com/peptamen-1-5-with-prebio1.html).

| Formula components | Urinary metabolites detected |
| --- | --- |
| Water | ND |
| Maltodextrin | ND |
| Enzymatically Hydrolyzed Whey Protein (from Milk) | Amino acids, Peptide |
| Medium Chain Triglycerides (from Coconut and/or Palm Kernel Oil) | Octanoyl glucuronide |
| less than 2% of Cornstarch | ND |
| Soybean Oil | ND |
| Soy Lecithin | ND |
| Magnesium Chloride | ND |
| Sodium Ascorbate | ND |
| Sodium Phosphate | ND |
| Calcium Phosphate | ND |
| Guar Gum | ND |
| Calcium Citrate | ND |
| Choline Chloride | Choline, Dimethylglycine |
| Potassium Chloride | ND |
| Salt | ND |
| Sodium Citrate | ND |
| Taurine | ND |
| L-Carnitine | Carnitine, Trimethyllysine |
| Magnesium Oxide | ND |
| Alpha-Tocopheryl Acetate | ND |
| Zinc Sulfate | ND |
| Ferrous Sulfate | ND |
| Niacinamide | Trigonelline |
| Calcium Pantothenate | Pantothenic acid |
| Vitamin A Palmitate | ND |
| Potassium Citrate | ND |
| Manganese Sulfate | ND |
| Pyridoxine Hydrochloride | Pyridoxic acid |
| Vitamin D3 | ND |
| Copper Sulfate | ND |
| Thiamine Mononitrate | ND |
| Riboflavin | ND |
| Beta Carotene | ND |
| Folic Acid | ND |
| Biotin | ND |
| Citric Acid | ND |
| Potassium Iodide | ND |
| Chromium Chloride | ND |
| Sodium Selenate | ND |
| Sodium Molybdate | ND |
| Phytonadione | ND |
| Vitamin B12. | ND |
| *ND: Metabolite/nutrient not detected when using MSI-CE-MS* |  |

**Figure S1.** Characterization of an unknown anion in urine subsequently identified (level 2) as propofol glucuronide based on high resolution MS (deprotonated molecular ion, [M-H]^-^ with most likely molecular formula) and MS/MS experiments demonstrating a neutral loss of a glucuronic acid (*m/z* 176.031) and formation of characteristic product ion as base peak in the spectrum (at 20 V), namely the deprotonated propofol anion (*m/z* 177.128). As an exogenous drug metabolite used as anesthetic during colonscopy, propofol glucuronide was subsequently excluded from the final metabolomics data matrix. This exclusion criteria was applied to all identified exogenous drugs as potential confounders, as well as infrequently (< 75%) and/or imprecisely (CV > 40%) measured endogenous metabolites excreted in urine and/or stool extracts.

**Figure S2.** Characterization of an unknown anion in urine subsequently identified (level 2) as phenylsulfate based on high resolution MS (deprotonated molecular ion, [M-H]- with most likely molecular formula) and MS/MS experiments demonstrating a neutral loss of a sulfur trioxide (*m/z* 79.961) and formation of characteristic product ion as base peak in the spectrum (at 20 V), namely the deprotonated phenol anion (*m/z* 93.0305). The urinary metabolite was identified with level 2 confidence in the absence of an authentic standard for unambiguous confirmation by co-migration and MS/MS spectral matching using same instrumental configuration.

**Figure S3.** Putative identification of an unknown organic acid anion in urine (level 3) that was significantly elevated in CD as compared to UC patients based on high resolution MS and MS/MS as shown for **(A)** an extracted ion electropherogram for the unknown anion (*m/z:*RMT:mode*,* 345.155:0.777:n) that is measured consistently in a representative run by MSI-CE-MS, **(B)** a high resolution TOF-MS spectrum showing its protonated molecular ion [M-H]^-^, charge state and isotope pattern required for determining its mostly likely molecular formula and **(C)** annotation of its MS/MS spectrum following collisional-induced dissociation (CID) at 20V of the precursor ion to confirm its likely chemical structure based on diagnostic fragment ions and neutral losses.


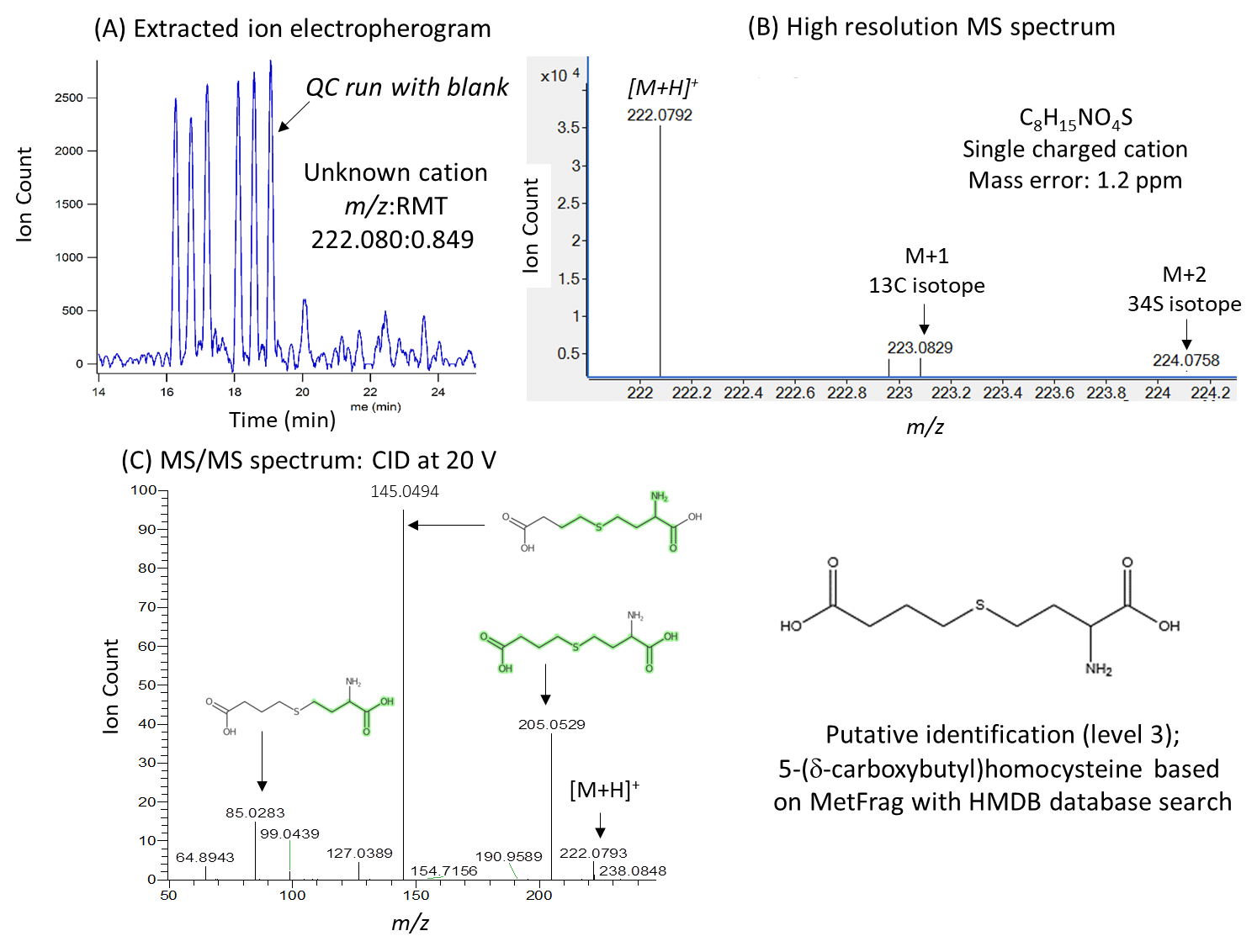


**Figure S4.** Putative identification of an unknown cationic/thiol derived metabolite in urine (level 3) that was significantly elevated in CD as compared to UC patients based on high resolution MS and MS/MS as shown for **(A)** an extracted ion electropherogram for the unknown anion (*m/z:*RMT:mode*,* 222.080:0.849:p) that is measured consistently in a pooled QC sample run by MSI-CE-MS without sample carry-over effects in blank, **(B)** a high resolution TOF-MS spectrum showing its protonated molecular ion [M+H]^+^, charge state and isotope pattern required for determining its mostly likely molecular formula and **(C)** annotation of its MS/MS spectrum following collisional-induced dissociation (CID) at 20V of the precursor ion to confirm its likely chemical structure based on diagnostic fragment ions and neutral losses.

**
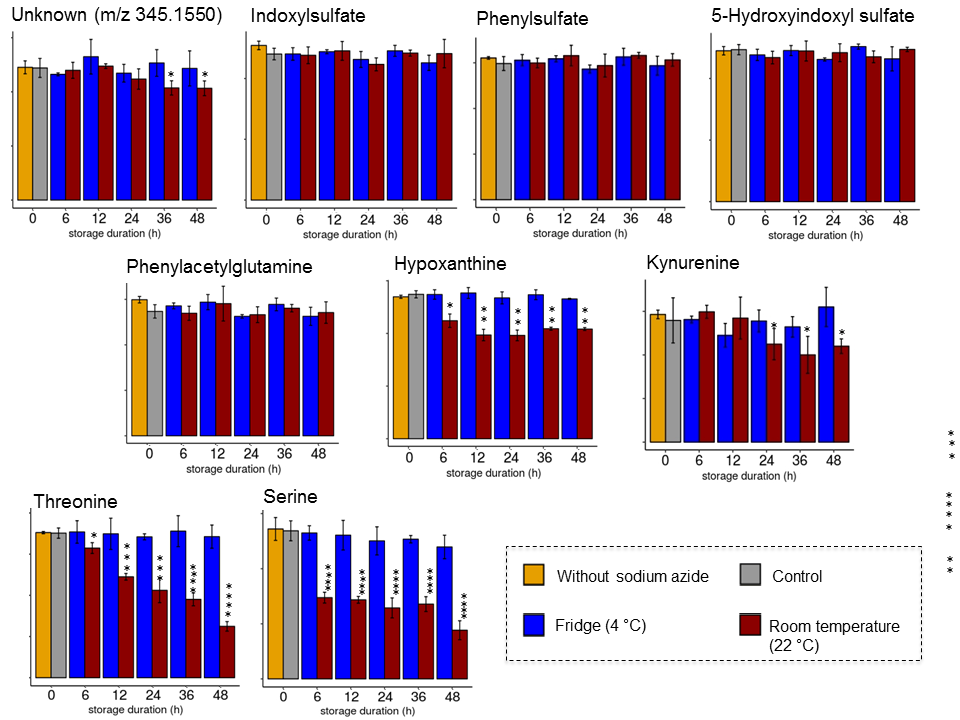
**

**Figure S5.** Representative plots illustrating the chemical stability of select urinary metabolites, where *, **, *** and **** indicate differences between control and each condition at *p* < 0.05, *p* < 0.01, *p* < 0.001, and *p* < 0.0001, respectively. A two-tailed homoscedastic student’s t-test was used for analysis, where y-axis represents relative peak area, and x-axis is storage duration over time.


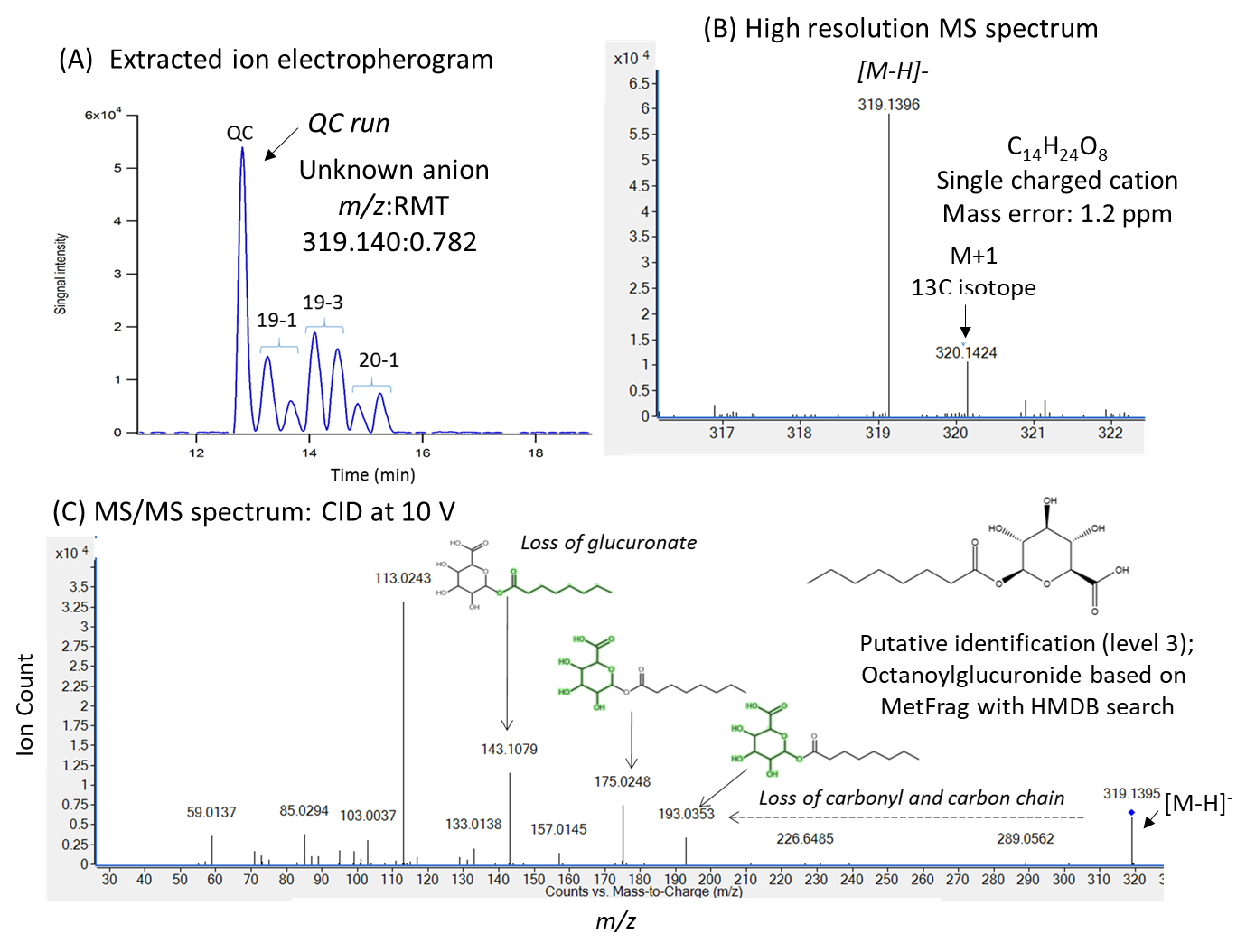


**Figure S6.** Putative identification of an unknown anion in urine (level 3) that was significantly elevated following EEN as compared to CS therapy in IBD patients based on high resolution MS and MS/MS as shown for **(A)** an extracted ion electropherogram for the unknown anion (*m/z:*RMT:mode*,* 319.140:0.782:n) that is measured consistently in a pooled QC sample run by MSI-CE-MS, **(B)** a high resolution TOF-MS spectrum showing its deprotonated molecular ion [M-H]^-^, charge state and isotope pattern required for determining its mostly likely molecular formula and **(C)** annotation of its MS/MS spectrum following collisional-induced dissociation (CID) at 10V of the precursor ion to confirm its likely chemical structure based on diagnostic fragment ions and neutral losses.

**
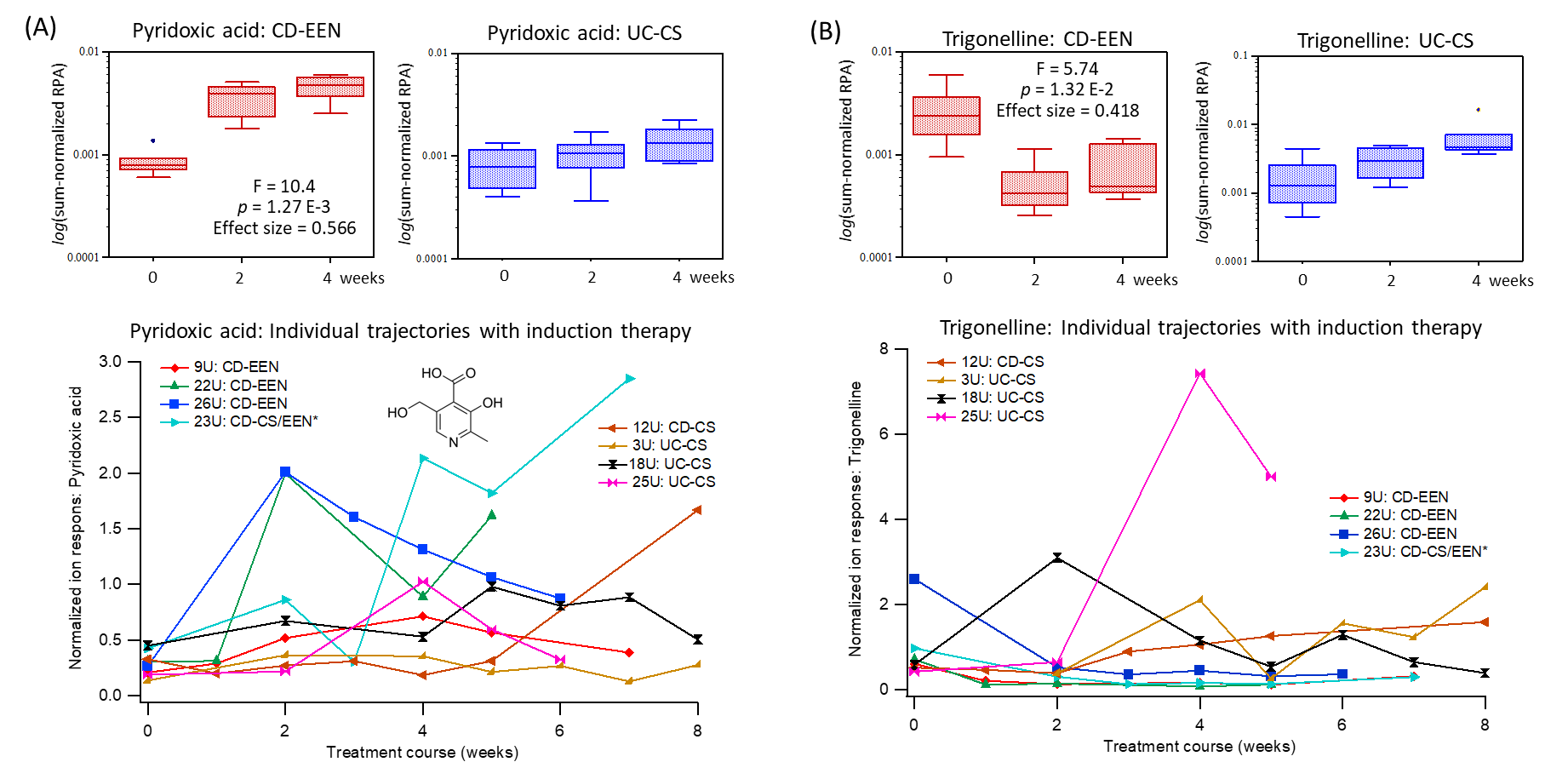
**

**Figure S7.** Secondary urinary biomarkers associated with adherence to EEN among pediatric IBD children (*n*=10), namely **(A)** pyridoxic acid and **(B)** trigonelline. Box-whisker plots show a specific yet more modest elevation in the excretion of both urinary metabolites following the initiation of ENN as compared to CS therapy at 2 and 4 weeks relative to baseline levels when using a repeat measures 2-way ANOVA with moderate to weak effect sizes. Also, metabolic trajectories for urinary octanoylglucuronide and pyridoxic acid are also shown for individual IBD patients in the two treatment arms (CD-EEN; UC/CD-CS, *n*=8) over the full 8 week intervention, including one CD patient who was later switched to ENN from CS after 2-3 weeks (CD-CS/EEN). For the latter patient, this clinical treatment course change is more evident in urinary excretion of pyridoxic acid than trigonelline.


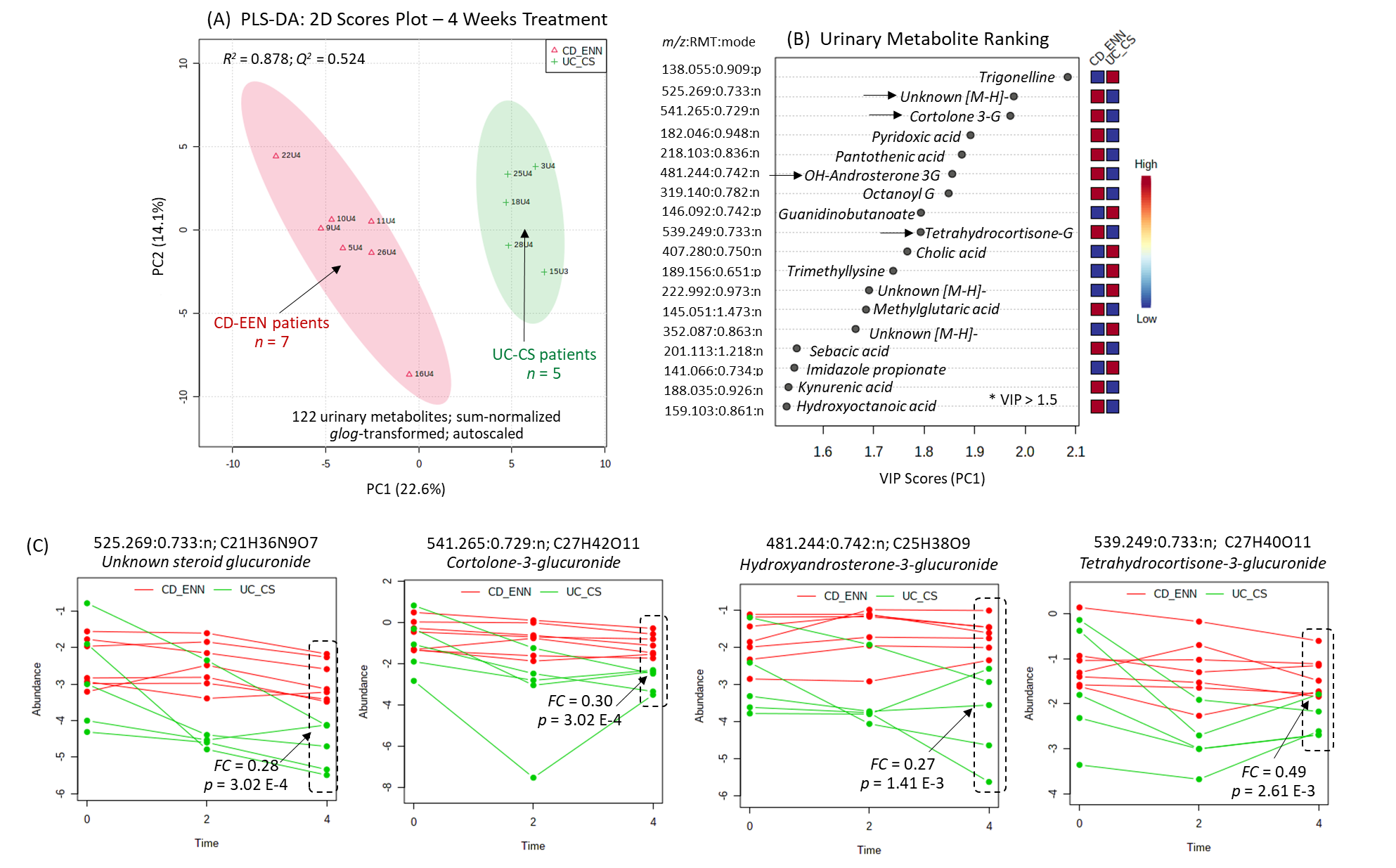


**Figure S8.** **(A)** Supervised multivariate data analysis of the urine metabolome from pediatric IBD patients after 4 weeks of EEN (*n*=7) or CS (*n*=5) therapy when using partial least squares-discriminate analysis (PLS-DA) that allows for differentiation of treatment regimes between IBD children based on **(B)** 18 top-ranked urinary metabolites having variable importance in projection (VIP) scores > 1.5. **(C)** Metabolic trajectories highlighting that several urinary corticosteroid conjugates were progressively lower following oral methylprednisolone treatment at 2 and 4 weeks as compared to UC baseline levels as well as the CD-ENN treatment arm.


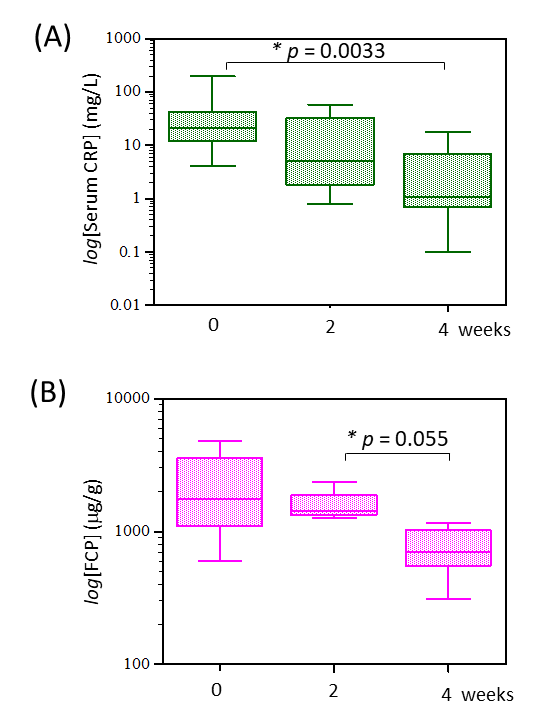


**Figure S9.** The efficacy of complementary induction therapy treatments to reduce active inflammation among pediatric IBD patients (CD-EEN, *n*=7; UC-CS, *n*=5) over an initial 4 week period from baseline. Serum CRP displayed a linear decrease with a significant reduction at 4 weeks (median CRP concentrations of 21, 5.1 to 1.1 mg/L) towards normal reference levels similar to stool derived FCP albeit with a longer lag time for induction (median FCP concentrations of 1800, 1432, and 703 μg/g). Stool specimens for FCP measurements however were less consistently collected from IBD affected children throughout this study once discharged from hospital.

**
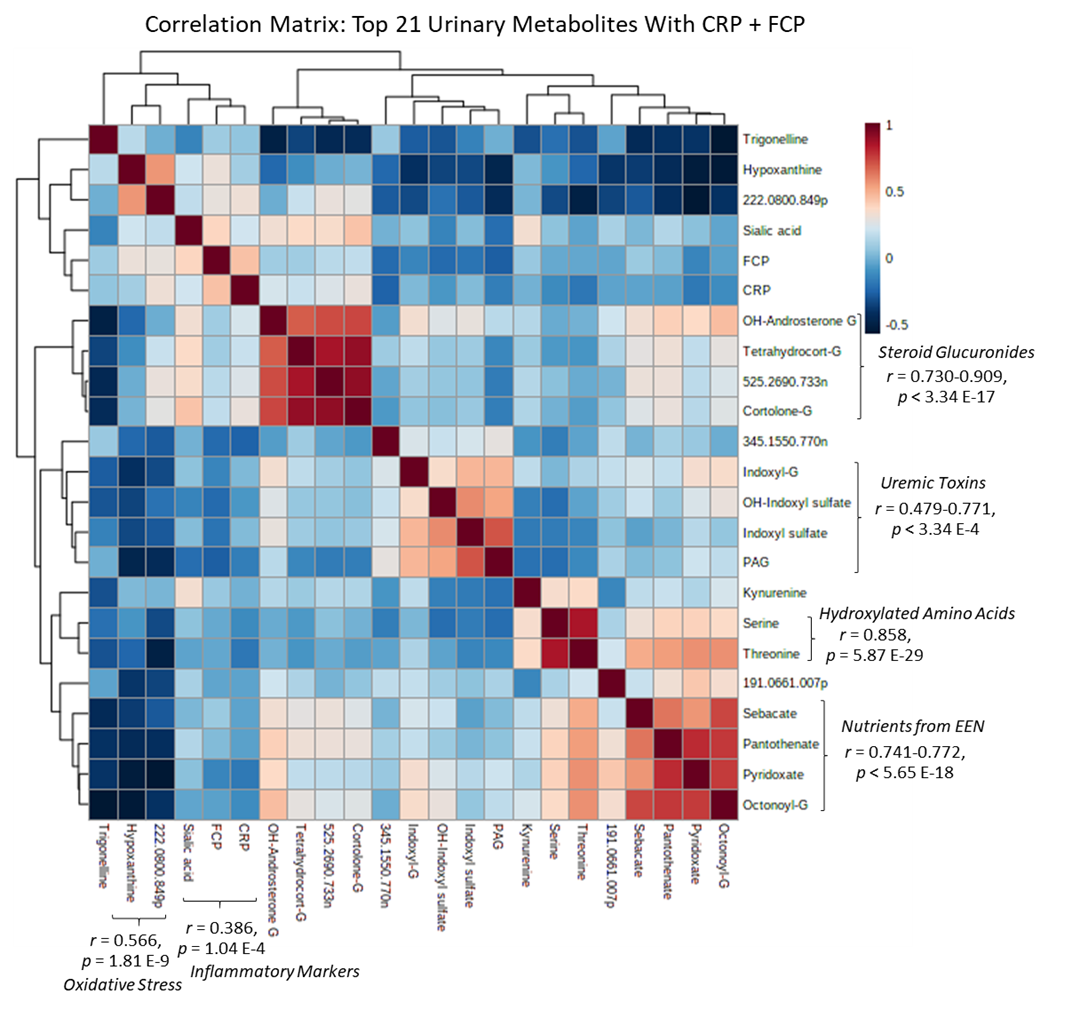
**

**Figure S10.** Correlation matrix (Pearson, *glog*-transformed, sum normalized) based on 21 urinary biomarkers associated with differentiation of pediatric IBD sub-types and treatment responses to EEN therapy identified in this work (repeat urine samples from all IBD patients over 8 weeks, n=96) in relation to serum CRP and FCP as classic biomarkers of systemic/colonic inflammation. Six major clusters of urinary metabolites are evident in this correlation matrix, including hydroxylated amino acids (*r* = 0.858), endogenous steroid glucuronide conjugates (*r* = 0.730-0.909), exogenous nutrients derived from EEN formula (*r* = 0.741-0.772), uremic toxins (*r* = 0.479-0.771), as well as urinary sialic acid with stool derived FCP (*r* = 0.386), and FCP with serum CRP (*r* = 0.442).
